# Kaleidoscopic imaging patterns of complex structures fabricated by laser-induced deformation

**DOI:** 10.1038/ncomms13743

**Published:** 2016-12-02

**Authors:** Haoran Zhang, Fengyou Yang, Jianjie Dong, Lena Du, Chuang Wang, Jianming Zhang, Chuan Fei Guo, Qian Liu

**Affiliations:** 1 Chinese Academy of Sciences (CAS) Center for Excellence in Nanoscience, CAS Key Laboratory of Nanosystem and Hierarchical Fabrication, National Center for Nanoscience and Technology, Beijing 100190, China; 2 Faculty of Electron and Materials, University of Chinese Academy of Sciences, Beijing 10080, China; 3 Suzhou Institute of Nano-tech and Nano-bionics, Chinese Academy of Sciences, Suzhou 212213, China; 4 Department of Materials Science and Engineering, South University of Science and Technology of China, Shenzhen 518055, China; 5 The MOE Key Laboratory of Weak-Light Nonlinear Photonics and TEDA Applied Physics Institute and School of Physics, Nankai University, Tianjin 300457, China

## Abstract

Complex surface structures have stimulated a great deal of interests due to many potential applications in surface devices. However, in the fabrication of complex surface micro-/nanostructures, there are always great challenges in precise design, or good controllability, or low cost, or high throughput. Here, we present a route for the accurate design and highly controllable fabrication of surface quasi-three-dimensional (quasi-3D) structures based on a thermal deformation of simple two-dimensional laser-induced patterns. A complex quasi-3D structure, coaxially nested convex–concave microlens array, as an example, demonstrates our capability of design and fabrication of surface elements with this method. Moreover, by using only one relief mask with the convex–concave microlens structure, we have gotten hundreds of target patterns at different imaging planes, offering a cost-effective solution for mass production in lithography and imprinting, and portending a paradigm in quasi-3D manufacturing.

## Abstract (web summary)

Complex surface micro- and nanostructures can be useful in many device applications, but are challenging in terms of controllability, low cost and high throughput. Here the authors have fabricated quasi 3D structures by the thermal deformation of simple two-dimensional laser-induced patterns.

## Graphical abstract

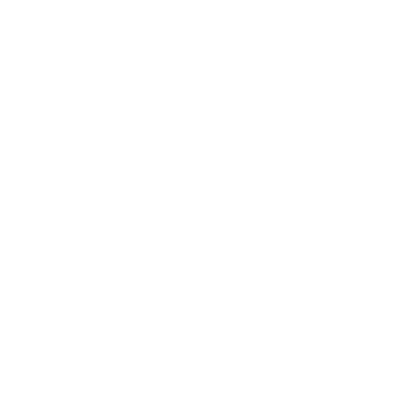

Various surface/interfacial micro/nanostructures have been widely used in solar cells^1^,^2^,^3^,^4^, light emitting diodes^5^, surface wetting and adhesion^6^,^7^,^8^, metamaterials^9^,^10^,^11^,^12^, cell biophysics^13^ and so forth. With the rapid development of surface/interface science or/and engineering, study on the fabrication of complex surface structures has been put on the agenda. Conventional micro/nanofabrication techniques such as photolithography and e-beam lithography are typically time consuming and costly to fabricate surface micro/nanostructures especially for those with a complex curved surface. Some unconventional methods, although can make surface structures at a low cost and high thoughput^14^,^15^,^16^,^17^,^18^,^19^,^20^,^21^,^22^,^23^,^24^,^25^, can often make only one specific structure, or have limited controllability and thus the fabricated structures exhibit deficiencies. For example, nanosphere lithography typically has limited size of hexagonal domains^26^. Metallization with an anodic aluminium oxide template can make a percolating nanofilm, but the size and the regulation are limited^27^. Grain boundary lithography can only be used to make curved nanowires, although it is capable of making large-area sub-100 nm nanowire networks of metals^28^. Microphase separation of block copolymers can be used to make nanoscale quasi-three-dimensional (quasi-3D) structures with curved surface, the height of the structure is typically only several nanometres, limiting its applications, and the structure could not be tailored to diverse shapes as well^29^. Various CaCO_3_ microlens arrays with a biomimetic smooth shape such as the brittle star, which can be fabricated by mineral precipitation self-assembly based on Aizenberg’s original discovery, have attracted the attention of scientists because of its simple fabrication process, although materials that can be utilized are limited^30^, ^31^, ^32^. Utilizing a two-dimensional (2D) pattern as the seed to form strain surface structures or quasi-3D structures^20^,^33^,^34^,^35^,^36^ is especially suitable for the fabrication of complex surface structures and naturally compatible with planar technologies, and this method has been thought to be one of the most promising emerging techniques, although some difficulties in designability and controllability are still remained. Recently, a wonderful work illustrated dozens of 3D structures created by combining conventional photolithography with a compressive buckling process^37^, displaying great potential for fabricating 3D structures by using planar patterns as the precursors.

Here we present a different route to fabricate various quasi-3D complex surface structures by introducing 2D laser-induced patterns (LiPs) as the precursors. Moreover, we demonstrate a multi-pattern imaging phenomenon similar to a kaleidoscope by using only one relief mask with the complex structures we fabricated.

## Results

### Laser-induced dot deformation and interaction among LiDs

**Figure 1:**
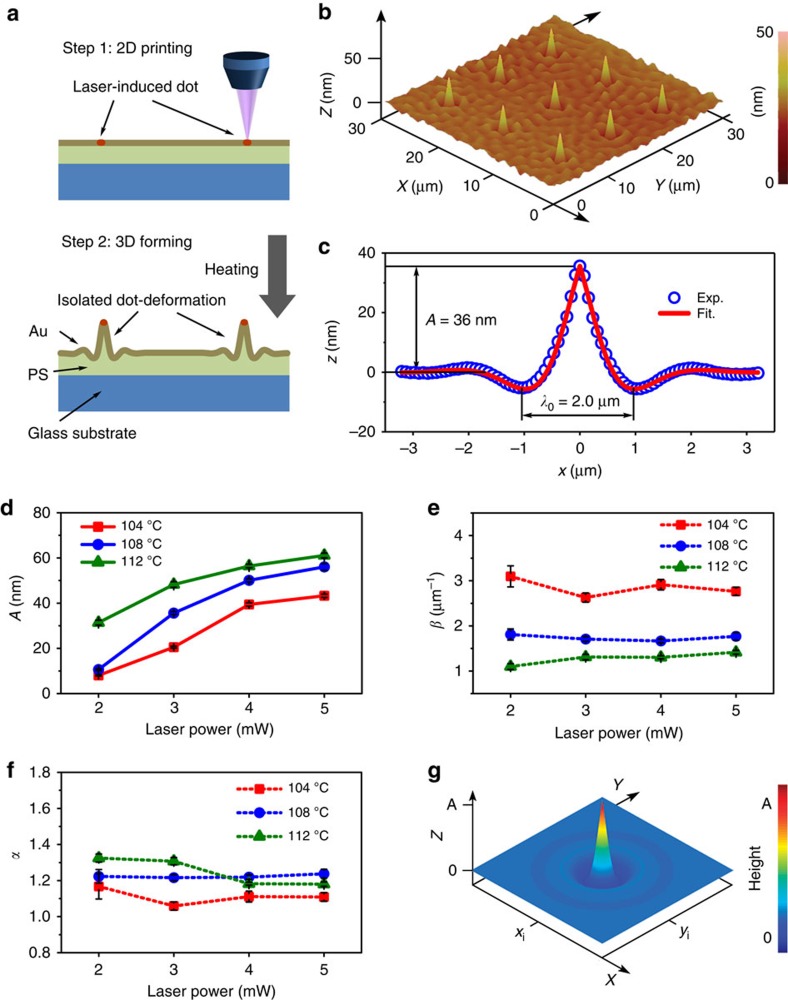
Laser-induced dot deformation. (**a**) Schematic for generation of isolated quasi-3D plastic dot-deformations. (**b**) AFM image of experimental dot deformations. (**c**) Experimental dot deformation section curve fitted by a damping function. (**d**) Amplitude (*A*) as a function of laser power and heating temperature. (**e**) Heating-temperature-dependent attenuation factor (*β*). (**f**) Correction factor (*α*) less dependent on laser power and heating temperature. (**g**) Visual LiDs. Error bars represent s.d.

Generally, when writing a dot using a laser single pulse on a bilayer consisting of a rigid capping layer and a soft underlying layer and then heating it to the glass-transition temperature (*T*_g_) of the underlying layer for about 10 min, a dot-like bump can generate exactly in the position of the dot. Here the laser-induced isolated dot acts as the seed for local deformation (Fig. 1a). An experimental result (Fig. 1b) demonstrates well uniformity of some isolated dot deformations in an Au (7 nm)/polystyrene (PS) (400 nm) bilayer, which is obtained under a laser pulse (3 mW, 150 ns) with a 300 nm spot size followed by heating at 108 °C, which is right above the *T*_g_ of polystyrene (PS, *T*_g_∼105 °C). Interestingly, the sectional curve of all the dot deformations measured by using atomic force microscopy (AFM) can well fit a damping function shown in Fig. 1c. That is, the dot deformation height (*Z*) at a distance (*x*) away from central position of the laser-induced dot can be described as *Z*(*x*)=*A* cos(2*πx*/*αλ*_0_)·exp(−*β*|*x*|). Here, *λ*_0_=2.0 μm is the intrinsic wavelength determined by the bilayer system^36^,^38^, and the amplitude *A*=36 nm, the correction factor *α*=1.2 and the attenuation factor *β*=1.7 μm^−1^ are determined by fitting with the experimental data. For a given bilayer system, *A, α* and *β* are related to the laser pulse power *P* (here we fix a pulse width of 150 ns for consistency) and the heating temperature *T*, as shown in Fig. 1d–f, and can always be determined experimentally (see ‘Methods’ section). Consequently, a typical laser-induced dot deformation (LiD) induced by a single pulse shown in Fig. 1g can be extracted as the basic surface deformation unit which can further form complex quasi-3D structures by the superposition of LiDs, and the profile height (*Z)* in arbitrary position (*x, y*) for a LiD located at (*x*_*i*_, *y*_*i*_) can be expressed mathematically as:


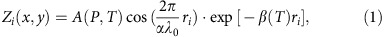


Where *r*_*i*_ *=*[(*x−x*_*i*_)^2^*+*(*y−y*_*i*_)^2^]^1/2^. The profile of the LiDs can be controlled by many factors such as *T*, *P* and the configuration of the bilayer system, implying a strong flexibility in design and fabrication.

When two or more LiDs are closer to each other, interaction among them will gradually intensify. Since LiD is a papillate deformation that can be described by a damping mechanical wave function, the final profile height (*Z*) at any position (*x, y*) can be expressed by a simple linear superposition of all LiDs:


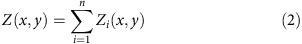


**Figure 2:**
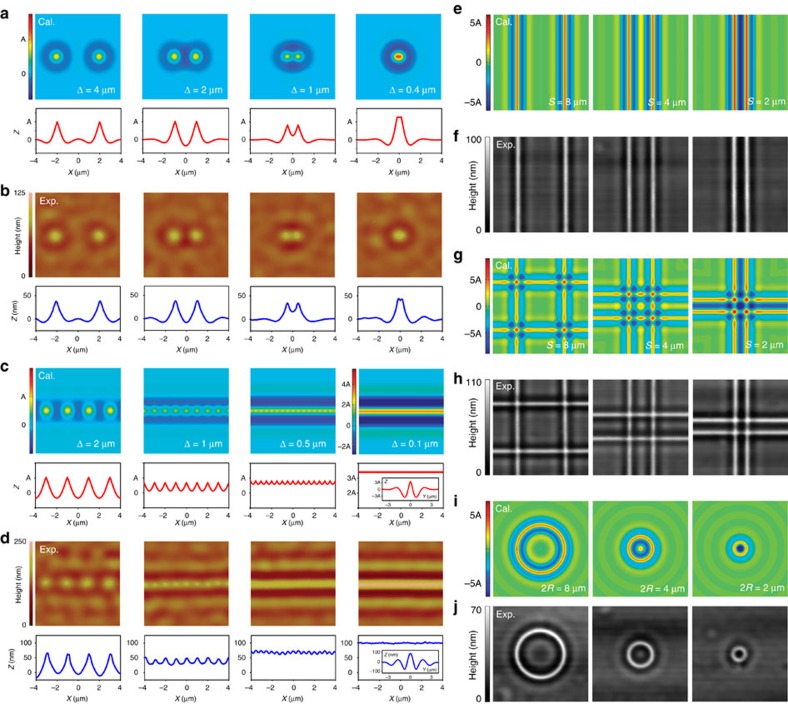
Superposition of LiDs. (**a**,**b**) Calculated and AFM experimental profile of two LiDs at different Δs. (**c**,**d**) Calculated and AFM experimental topographies and cross-sectional profiles of a row of LiDs for different Δs. (**e**,**f**) Calculated and laser scanning confocal microscope topographies of two parallel lines for different spaces (*S*). (**g**,**h**) Calculated and laser scanning confocal microscope topographies of four orthogonal lines for different *S*. (**i**,**j**) Calculated and laser scanning confocal microscope topographies of circles for different radius (*R*). Image size in the left (right) column is 8 μm × 8 μm (16 μm × 16 μm).

**Figure 3:**
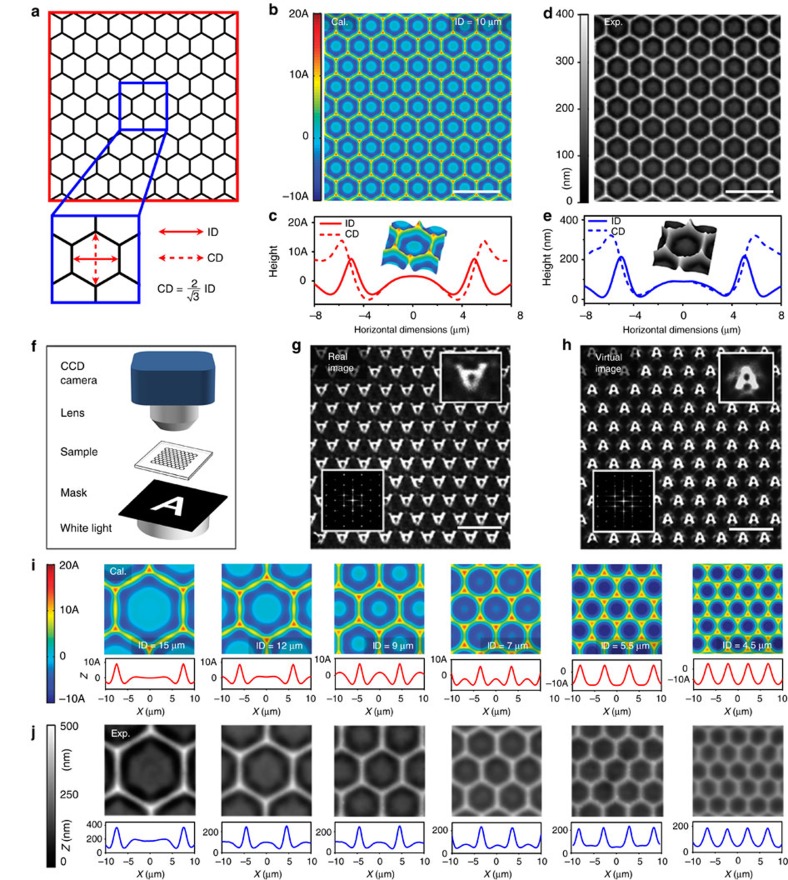
Design and fabrication of convex–concave microlens array. (**a**) Schematic of a planar honeycomb array as a 2D LiP. (**b**) One region with 80 μm × 80 μm in a large-area of calculated convex–concave microlens array. (**c**) Calculated 3D topography of an individual cell showing fine distinction in the circumscribed circle diameter (CD) and ID. (**d**,**e**) Experimental results corresponding to panels (**b**,**c**) respectively, under conditions of a laser pulse power of 4 mW (150 ns), heating temperature of 108 °C. (**f**) Schematic for the imaging test of a convex–concave lens array. (**g**,**h**) Real and virtual images of the letter A by using the convex–concave lens array, respectively. (**i**,**j**) Calculated and experimental topographies of microlens arrays with the same area of 20 μm × 20 μm but different ID of 15, 12, 9, 7, 5.5 and 4.5 μm, respectively. Here the experimental topographies are obtained by using a laser scanning confocal microscope. Corresponding scanning electron microscopy images show that the surface of the lenses is smooth in Supplementary Fig. 5. Scale bars, 20 μm.

As verification to equation (2), we first calculated the surface profile of two adjacent LiDs with different intervals (Δs), as shown in Fig. 2a. It shows that the final profiles at different Δs differ in height and waveform, agreeing well with our experimental results (Fig. 2b). When several LiDs with the same interval Δ were aligned in a straight line (Fig. 2c), the calculated morphologies vary with a decrease in Δ and the profile transforms from a ripple to a flat line as Δ reduces to 0.1 μm, agreeing well with our experiment again (Fig. 2d). Curves and lines hereinafter refer to the case that Δs≤0.1 μm unless stated. In addition, both the calculated and experimental cross-section of a line in Fig. 2c,d are similar to that of a LiD (Fig. 1c). It should be noted that the main line structure is significantly heightened to about 180 nm in the inset of the bottom right corner in Fig. 2d (depth–width radio of ∼1:11, two times larger than that of a single LiD), and the height of 280 nm (depth–width ratio of 1:5) or even higher value can be reached (Supplementary Fig. 1). Surface structures with such a height have been proven to be able to employ as a hydrophobic surface (Supplementary Fig. 2), microlens (Fig. 3), solar cells^1^,^2^,^3^,^4^ and light emitting diodes^5^. More interestingly, sub-structures on both sides of the main line become more featured due to the interaction of LiDs. In fact, the line structure can also be modified into many other waveforms by simply adjusting the height of each LiD, further demonstrating that the surface profile can be well controlled. For example, a slope structure is available by changing the laser power along the line (Supplementary Fig. 3). Figure 2e,f and Supplementary Fig. 4a,b show that the middle sub-structure generates as the space (*S*) between two lines approaches to 2*λ*_0_ and disappears as *S* reduces to *λ*_0_ or a smaller value, and this regulation is also applicable to closed lines or curves (Fig. 2g–j, Supplementary Fig. 4c–f). Note that except for height deviation caused by the limited strain capacity for a practical bilayer, the morphologies (Fig. 2f,h,j) are consistent well with calculated surface profiles (Fig. 2e,g,i). We believe that the superposition of the tunable LiDs with substructures enables the design and the formation of various complex quasi-3D surface structures. It should be stressed that our method is capable of fabricating various complex surface structures by using planar patterns which act as the seeds for surface structures, according to equation (2). This illustrates an interesting fact: we are able to use simple 2D patterns to generate complex surface or quasi-3D structures via a thermal straining processing, and this process is especially suitable for making structures with a smooth curved surface (Supplementary Fig. 5). However, according to equation (2), our method may not be desirable for making structures with sharp edges, and therefore it has restrictions in making sharp-edged structures in practical applications. Regarding this point, our method is similar to interference lithography but has a simpler process as well as a stronger capacity in designing different configurations.

### Design and fabrication of a complex structure

To demonstrate the ability to fabricate quasi-3D complexities, we selected a common hexagonal honeycomb pattern with an inscribed circle diameter (ID) of 10 μm (Fig. 3a) as a simple 2D LiP, as shown in Fig. 3a. A hexagonal lens array (Fig. 3b) can be figured out according to equation (2) on Au (25 nm)/PS(1,000 nm) bilayer with *λ*_0_=4.5 μm (see ‘Methods’ section). In the array each hexagonal cell consists of an annular concave lenslet around a circular convex one with a diameter of about 7 μm and six horn-like small humps located at the six vertexes of each hexagon, as illustrated in the inset of Fig. 3c. We call such array with the complex structure coaxially nested convex–concave microlens array. The size of the convex lenslet is close to the smallest clear-imaging lens in visible region^32^. Although the profile exhibits a small difference in the circumscribed circle diameter and the ID as shown in Fig. 3a, the outside concave lens and the embedded convex lens have approximate spherical surface allowing for clear imaging (Fig. 3c). Figure 3d,e shows the topology of a region with a size of 80 μm × 80 μm as well as that of an individual lens, displaying the consistency of the design and the experiment. It should be emphasized that to our best knowledge the complex coaxially nested convex–concave microlens array was not available before. In fact, the formation of a coaxially nested convex–concave microlens is a complex straining process, for which the first-order structure preferentially grows along the LiPs, and the secondary-order structures form convex–concave microlenses in the middle. Both clear real and virtual images of a letter A by the convex–concave microlenses have the same size and similar brightness (Fig. 3f–h), indicating good imaging performance and further proving the smooth surface and uniformity of the convex–concave microlenses (also see fast Fourier transform (FFT) patterns inserted in Fig. 3g,h).

We have also demonstrated that the shape, size and height of the as-made structures are well tunable. From the comparison of experimental and calculated morphologies and cross-sections illustrated in Fig. 3i,j, the imbedded secondary-structure convex lenses shrunk and flatten gradually and eventually vanish with the decrease of ID. With ID changing from 9 to 4.5 μm, the convex–concave microlens transfroms to a pure concave lens with clear-imaging capacity (Supplementary Fig. 6), and more complex structures forms for a ID of 15 and 12 μm, which are larger than 2.5 *λ*_0_. Such a transition (cross-sections in Fig. 3j) is because the LiPs can well define the strain distribution of the bilayer, which can also be predicted by the superposition of LiDs (cross-sections in Fig. 3i). The shape of the microlenses can also be diverse geometries rather than regular hexagon only, for example, elongated hexagons, squares and combined shapes (Supplementary Fig. 7). Besides, the high controllability allows us to easily adjust the height of the microlens from ∼25 nm to ∼150 nm for the same aperture diameter (Supplementary Fig. 8), or retain the shape and curvature unchanged for different aperture diameters from several microns to about 800 nm (Supplementary Fig. 9). It is easy to see from Supplementary Fig. 9b that the resolution limit of the surface structure is about 300 nm (minimum full-width at half-maximum), which depends mainly on the laser spot size.

To make 3D, or quasi-3D, or curved surface structures, conventional microfabrication methods including photolithography often require repeated process with many steps such as mask making, pattern transfer, development, fixing, etching and so on. By contrast, our method only has two simple steps: laser writing and heating. Moreover, because of no wet process involved, this method exhibits less defects and contamination compared with conventional photolithography. From another viewpoint, our method neither removes nor adds materials; therefore it is a green route. Conventional top-down fabrication method often involves in a process that part of the materials is removed, and 3D printing technique involves in adding materials from a nozzle. In addition, our method allows for large-area fabrication of surface structures (Supplementary Fig. 10), and the structures can be accurately replicated to a transparent or rigid material as a mask for imprint lithography or photolithography (Supplementary Fig. 11). This makes mass production of our structures possible.

### Kaleidoscopic imaging patterns

**Figure 4:**
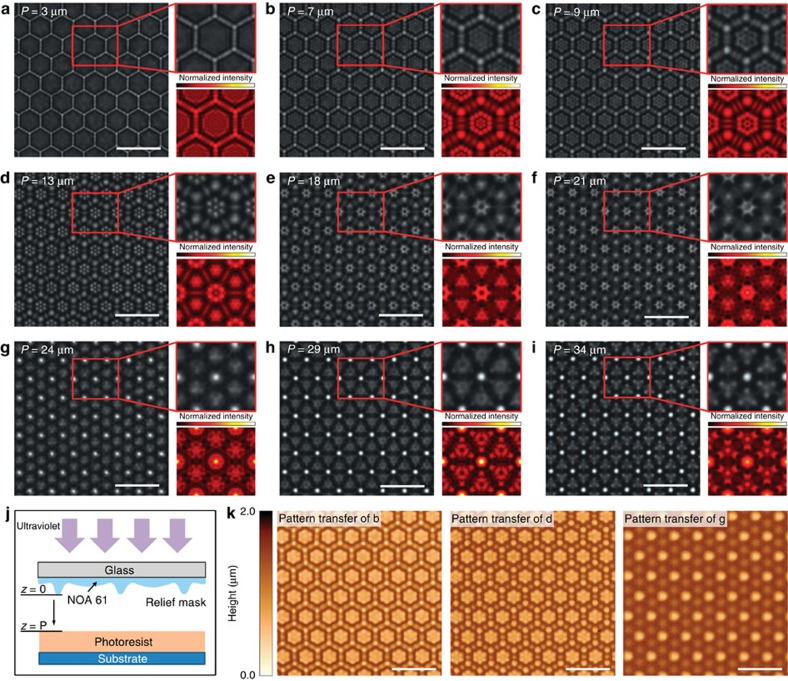
Kaleidoscopic images formed by a relief mask with a convex–concave microlens array. (**a**–**i**) Optical microscope-captured images by using a transparent convex–concave microlens array of NOA 61, with different imaging positions shown in **j** (*P*=3, 7, 9, 13, 18, 21, 24, 29 and 34 μm). (**j**) Schematic diagram of proximity-mode ultraviolet photography, with the convex–concave microlens array as a relief mask. (**k**) Transferring patterns in **b**,**d**,**g** on photoresist. Scale bars, 20 μm.

Various conventional masks have one common feature that they can often generate only one target pattern, so that users need masks with different patterns for different structures, and sometimes even a series of masks for complex structures. In recent years, some masks^39^,^40^ with periodic patterns have been developed for the fabrication of periodic 3D nanostructures based on Talbot effect, which refers to that one periodic structure under the illumination of a monochromatic plane wave or sphere wave can be reproduced with the same or scaling up period at some certain locations behind the periodic structure. Such masks promote 3D fabrication through several Talbot images at different planes in a thick photoresist, but Fresnel images between the Talbot images are limited and monotonous because of the single period structures of the mask. Here we demonstrate an interesting relief mask with our convex–concave lens array, which is quite different from the masks with single period structures. Our mask can realize richer imaging patterns at different imaging planes just like a kaleidoscope (Fig. 4a–i, Supplementary Movie 1), which inspires an interesting hypothesis—one mask can be used to generate multi-pattern diffraction images which not only locate at the Talbot distances or fractional Talbot distances, but also at other distances, only by simply changing the distance between the mask and the photoresist. In Fig. 4j,k, we verified this hypothesis and successfully transferred different patterns to photoresist (AR-P 3110) using ultraviolet lithography (365 nm). Our simulation has indicated that the relief structure (or the height) significantly affects the kaleidoscopic imaging (Supplementary Fig. 12). This mainly stems from the fact that the relief mask along its height direction can be divided to many sections with different structures, which will result in kaleidoscopic imaging due to the superposition of multitudinous optical-field distributions at the same distance between the mask and imaging plane. The relief structures help generate richer structures than common masks in photolithography. We here proposed an interesting mask that is called the kaleidoscopic mask as a powerful tool to create abundant target patterns especially those with a smoothly curved surface for lithography by using only one complex quasi-3D mask.

In summary, we have presented a fabrication method for diverse quasi-3D complex surface structures by heating a 2D pattern. We have shown that the structures are well designable and can be controllably fabricated in a simple and cost-effective way. The formation of the structures can also be predicted by using simulations, which can be well consistent with the structure profiles in experiment. Large-area fabrication capacity and replication technique elucidated by us have indicated a high compatibility for mass production. The kaleidoscopic mask provides a useful tool to create plentiful target patterns in photolithography by using only one mask. This might offer an avenue to solve the rocketing price of the mask in industry due to developing trend of product individuation and demands of more and more complex structures. We believe that our work will be helpful to fabricate more high-quality and functional quasi-3D structures especially complex structures which are otherwise quite expensive by using conventional fabrication techniques. This may motivate some applications of surface/interface with microscale structures in such as multi-signal sensors, 3D integral imaging, functional surfaces, and integrated optics devices especially for those with a smooth and curved surface, and may greatly promote surface structure applications in energy, photonics, metamaterials, biomedicine and so forth.

## Methods

### Fabrication process

A PS layer (thickness 300–1,000 nm) was first spin coated on a clean glass substrate using toluene solution of PS and was then heated at 60 °C for 12 h to remove residual solvent and to relieve stress. Then a Au layer was deposited (thickness 5–25 nm) on the PS layer by ion sputtering (Hitachi E-1010). After the Au/PS bilayer was prepared, a laser direct writer system (HWN LDW-P1500, laser wavelength 405 nm, laser spot size∼300 nm, pulse width 150 ns and laser power 1–5 mW) was used for writing various 2D LiPs on the bilayer. Corresponding quasi-3D surface structures were obtained by heating the bilayer with 2D LiPs at 108 °C (slightly over the glass-transition temperature of PS, ∼105 °C) in a vacuum oven for about 10 min. For LiPs on the bilayer, it exhibits a lower modulus compared with other unwritten regions, resulting in preferential deformation along LiPs.

### Design of 2D LiPs

Two-dimensional LiPs can be designed as dots, dotted line, straight line, parallel lines, vertical lines, circles, arbitrary curves, honeycomb-like hexagonal array or any other complex configurations. To ensure the equal-height structure and to improve the control precision, all the LiPs in experiment and in simulations were designed with a pixel pitch of 0.1 μm, and then were written in the bilayer by a laser direct writer (HWN LDW-P1500).

### Selection of bilayer systems

LiDs, as well as various quasi-3D structures grown from the 2D LiPs, are essentially a kind of strained structure in bilayer systems caused by compressive stresses. Strained structure has an intrinsic wavelength (*λ*_0_) depending on the elastic-moduli and thicknesses of the two layers. Both in calculation and experiment, we all need to select suitable bilayer system with a *λ*_0_ matching the design. In our work, four different Au/PS bilayer systems were used (System 1: Au (7 nm)/PS (400 nm), *λ*_0_=2.0 μm; System 2: Au (25 nm)/PS (1,000 nm), *λ*_0_=4.5 μm; System 3: Au (5 nm)/PS (300 nm), *λ*_0_=1.7 μm; System 4: Au (10 nm)/PS (600 nm), *λ*_0_=2.9 μm). System 1 was used to study single LiD and the superposition of LiDs. We also fabricated several quasi-3D structures based on System 1. System 2 with a larger *λ*_0_ was used to fabricating the convex–concave lens array and pure convex lens array. Systems 3 and 4 were used to show the controllability of aperture size with the shape and curvature unchanged (Supplementary Fig. 9). System 4 was also used to make large-area convex–concave lens arrays.

### Determination of parameters

For a certain bilayer system, parameter *A, α* and *β*, are dependent on laser power (*P*) and heating temperature (*T*). They can be determined by fitting the experimental profile of LiDs. First, we made several LiDs on a certain condition (Pi,Ti), and measured the average profile of them. And then we used a damping function, *z*(*x*)=*A* cos(2*πx*/*αλ*_0_) exp(—*β*|*x*|), to fit the average profile mathematically and finally obtained one set of parameters (*A*_*i*_*, α*_*i*_*, β*_*i*_) corresponding to the condition (*P*_*i*_*,T*_*i*_). Next, we analysed the dependence of *A, α, β* on *P* and *T*, as shown in Fig. 1d–f. With the increase of *T, A* increases due to the increase of compressive thermal stress; while with the increase of *P*, *A* also increases due to the fact that stronger laser pulse leads to a greater softening effect on Au film (decrease of modulus). Attenuation factor *β* is almost independent on *P*, but declines with the increase of *T* because PS becomes more viscous at higher temperatures. Correction factor *α* shows a weak dependence on both *P* and *T*, and could generally be considered as a constant.

### Characterization

The morphologies and cross-sections of quasi-3D structures were measured by an atomic force microscope (AFM, Veeco, Dimension 3100) or/and a laser scanning confocal microscope (LSCM, Olympus, LEXT-OLS4000). The transmittance spectrum of Au/PS and NOA 61 was recorded by using a ultraviolet–vis–NIR spectrometer (Shimadzu, UV-3600). Tilted scanning electron microscopy images in Supplementary Fig. 5 were capured by FEI-Nova-200 nanoLab.

### Replication and transfer

For a reproducible soft template of various surface quasi-3D structures fabricated on Au/PS, polydimethylsiloxane (PDMS) was prepared by mixing the elastomer base and curing agent (Sylgard 184, Dow Corning) at a volumetric ratio of 10:1 and poured onto the structured bilayer followed by curing at 60 °C for 2 h in a vacuum oven. Then, the cured PDMS moulds were detached from the bilayer and used for soft contact printing. Next, Norland Optical Adhesive (NOA 61, refractive index ∼1.56 for the cured polymer) was dropped onto the structured surface of PDMS mould, and the target substrate (glass slice for instance) was placed on top without applying any additional pressure. After curing under ultraviolet light for 5 min (365 nm, 200 mW cm^−2^), the PDMS mould was removed from the quasi-3D structures on the target substrate (Supplementary Fig. 11).

### Capture of kaleidoscopic images

Imaging experiments of the convex–concave lens array were performed by using an optical microscope (Leica DM 2500) with a white light source. For capturing kaleidoscopic images generated by the convex–concave lens array, above all, we placed the sample with a quasi-3D structure upward on the sample stage of the microscope, then illuminating it with a white light source from below. Next, we adjusted the vertical position of the sample stage until we observed clear image of the convex–concave lens array. At this time, the focal plane of the objective lens coincided with the sample surface. In other words, the distance between the focal plane of the objective lens and the sample surface, which we defined as imaging distance, was zero at the setpoint, or *P*=0. Afterwards, we gradually increase *P* from 0 to more than 200 μm and capture images every micron. Finally, we organized these images according to imaging distance into a video (Supplementary Movie 1) to show the whole kaleidoscopic evolution.

### Ultraviolet lithography

To further confirm the kaleidoscopic phenomenon, we transferred several patterns to a positive photoresist (AR-P 3110, Allresist) by using a double sided mask aligner system (Karl Suss, MA6) with a 365 nm ultraviolet light. In this experiment, it should be noted that deviation existed in the same imaging distance owing to the different wavelengths of white light and ultraviolet light.

### Numerical simulations

The finite-difference time-domain (FDTD) method was used to simulate electromagnetic wave propagation in investigated structures. For the periodic structure, the FDTD simulations were performed in its unit cell (20 μm × 17.3 μm) and periodic boundary conditions were used at side walls of the unit cell.

### Data availability

All the data that support the findings of this work are available from the corresponding authors upon reasonable request.

## Additional information

**How to cite this article:** Zhang, H. *et al*. Kaleidoscopic imaging patterns of complex structures fabricated by laser-induced deformation. *Nat. Commun.* **7,** 13743 doi: 10.1038/ncomms13743 (2016).

**Publisher’s note**: Springer Nature remains neutral with regard to jurisdictional claims in published maps and institutional affiliations.

## Supplementary Material

Supplementary Information Supplementary Figures 1-12

Supplementary Movie 1
