# Supplementary material for: Kaleidoscopic imaging patterns of complex structures fabricated by laser-induced deformation
Source: Nat Commun. 2016 Dec 2;7:13743. doi: 10.1038/ncomms13743 (PMC5476795; doi:10.1038/ncomms13743)
Supplement: Supplementary Information — Supplementary Figures 1-12 [file ncomms13743-s1.pdf]

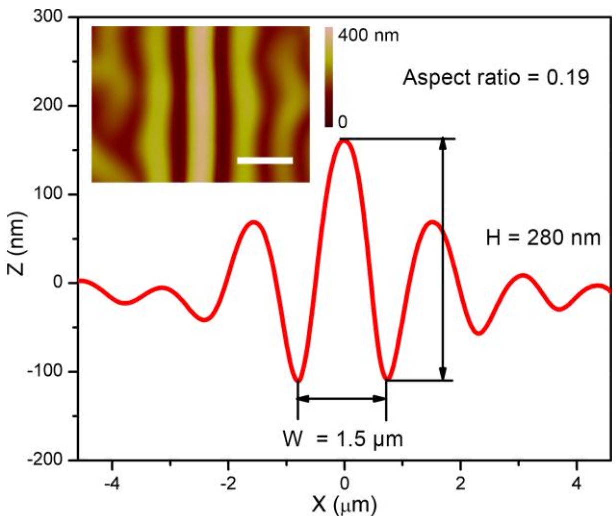

Supplementary Figure 1 | Depth-width ratio. A line structure with height of 280 nm and width of 1.5 μm can be obtained by heating the bilayer at 140 ℃ for several hours, which relies mainly on the enhancement of the thermal stress level. Such depth-width ratio is about 1:5 for the Au (7 nm)/PS (400 nm) bilayer, which can be adjusted to a larger value by changing bilayer system including material types and their thicknesses. The scale bar is 2μm.

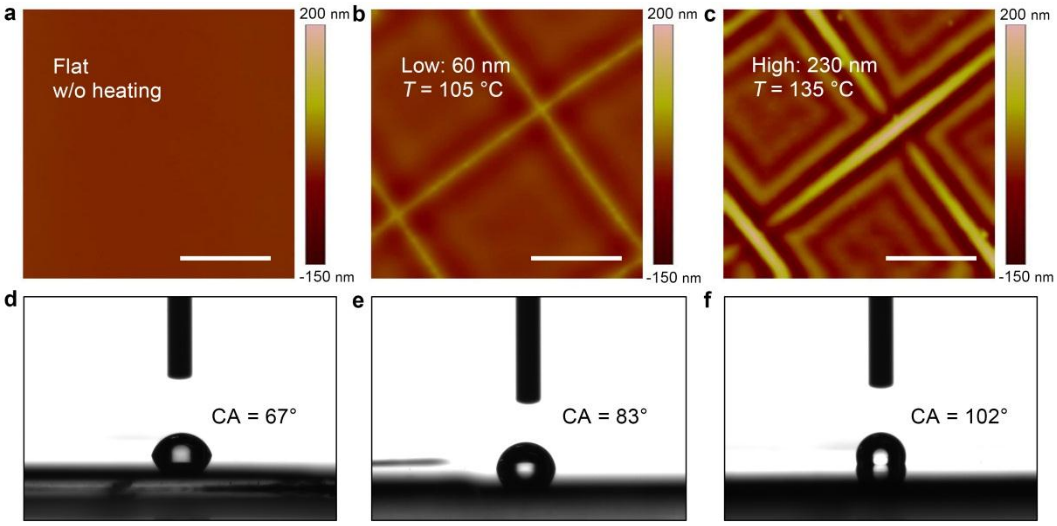

Supplementary Figure 2 | Wetting-tunable surface. a-c, Local AFM images of three different surfaces on Au/PS bilayers: (**a**) a flat surface without heating, (**b**) a 60 nm-high grid (spacing of 10 μm) structure obtained at a heating temperature (*T*) of 105 ℃, (**c**) and a 230 nm-high grid (spacing of 10 μm) structure obtained at a heating temperature (*T*) of 135 ℃. The scale bars are 5 μm. **d-f,** Water contact angle (CA) of corresponding surfaces, showing the modification of wetting property by the structure height. It should be stressed that the wetting property can be further improved by structural optimization.

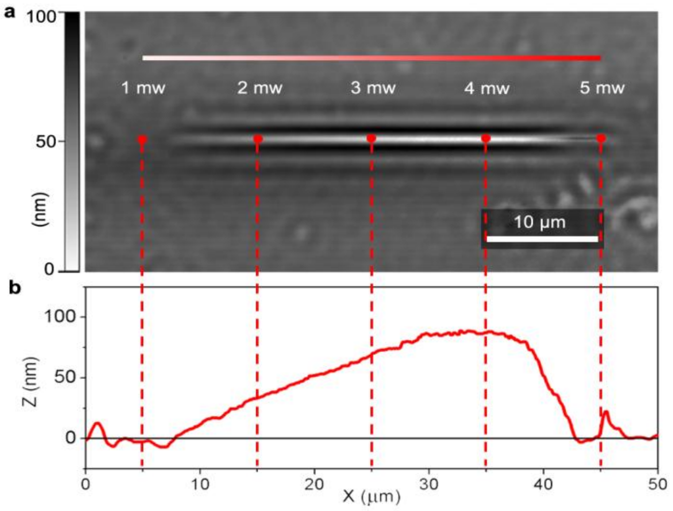

Supplementary Figure 3 | Slope-line structure. a, Morphology of a slope-line structure realized in experiment, where the power of writing laser along the straight line increases linearly from 1 to 5 mw. This height image was measured by laser scanning confocal microscope. **b,** Height profile of the slope structure, showing that the height of the structure increases linearly with the increase of the laser power in the range of 1.5 to 4 mW.

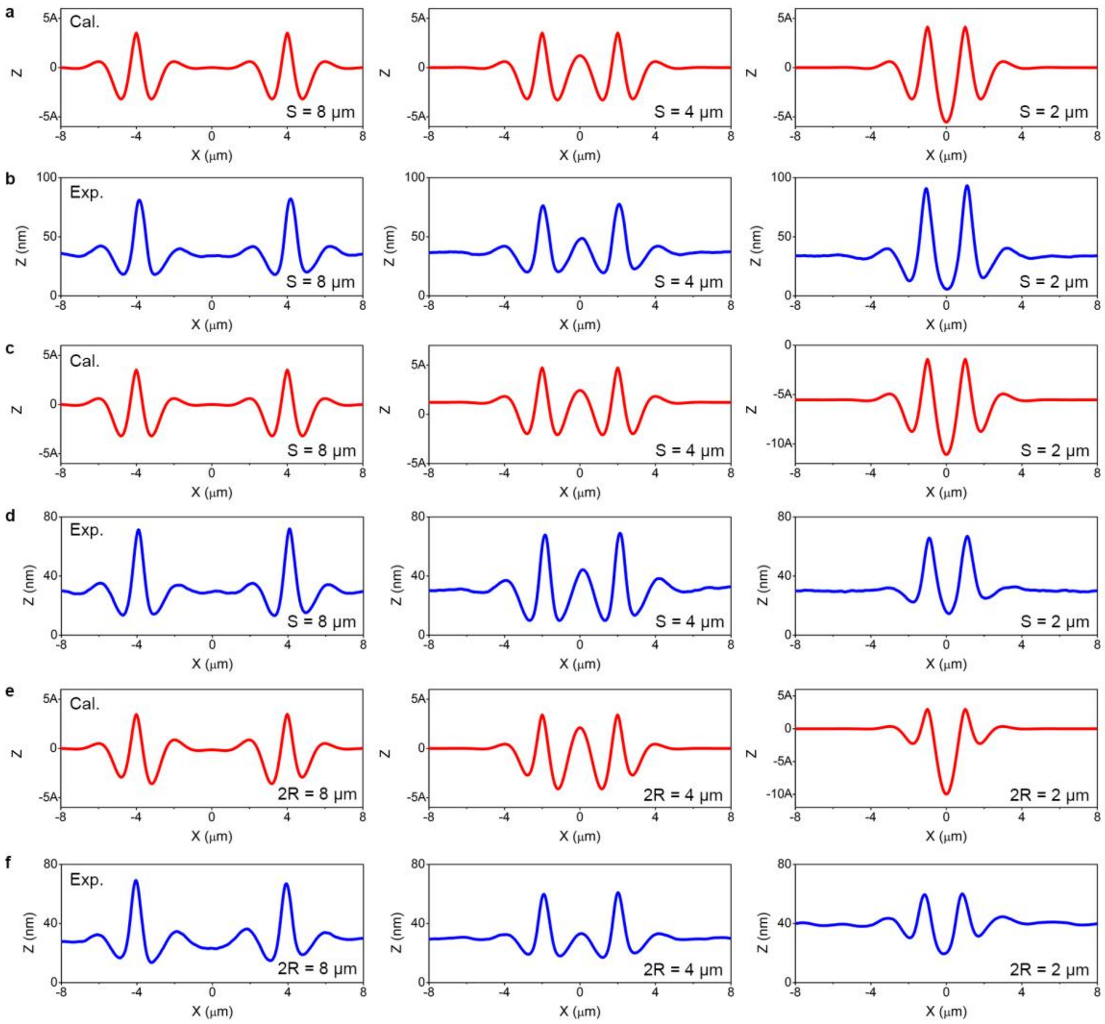

Supplementary Figure 4 | Tunable sub-structures. a,b, Calculated and experimental cross-sections of two parallel line-structures (Fig. 2e,f) with different space (*S*). **c,d,** Calculated and experimental cross-sections through center of square structures consisting of two sets of vertically aligned lines (Fig. 2g,h) with different space (*S*). **e,f,** Calculated and experimental cross-sections through the center of a circle (Fig. 2i,j) with different radius (*R*).

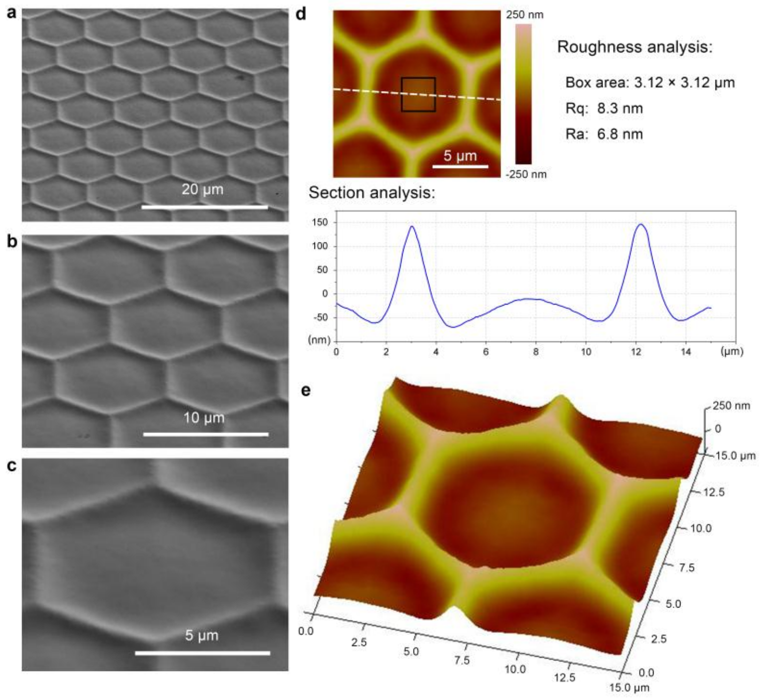

Supplementary Figure 5 |Surface roughness analysis of convex-concave microlens array. a-c, Tilted SEM images at different magnifications of a 9 μm-aperture convex-concave microlens array, showing the smoothness of the surface. **d,** AFM analyses of roughness and section of one microlens. Here the root-mean-square roughness is on the order of 10 nm, showing that the surface of the lens is smooth. The images are obtained with a new AFM tip with a radius of 8 nm (Bruke, RTESP-300). **e,** Topographic AFM height image of one microlens.

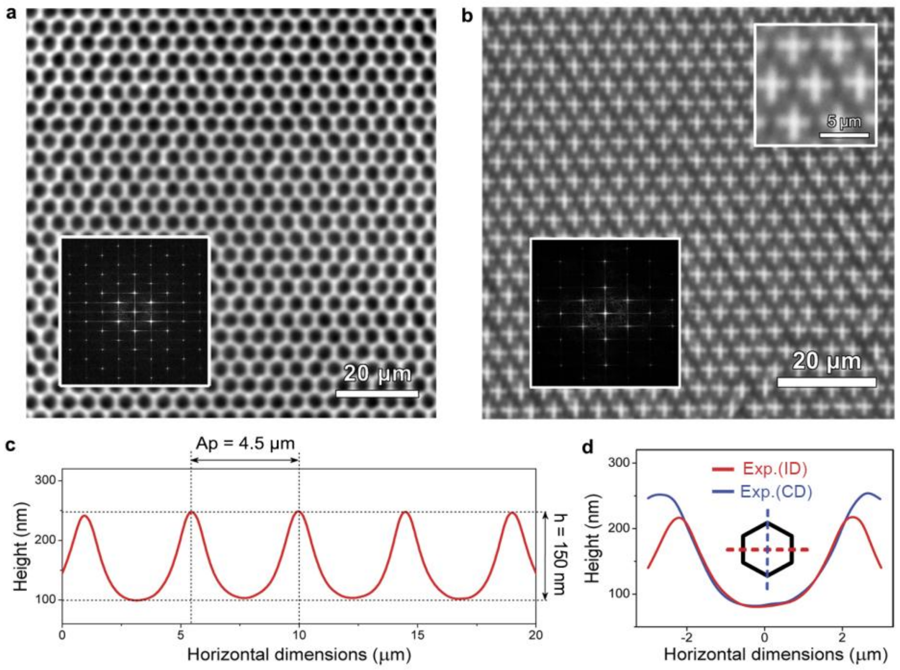

Supplementary Figure 6 | Pure concave microlens array with an aperture (Ap) size of 4.5 µm.

a, Topography of the pure microlens array (obtained by a laser scanning confocal microscope) and corresponding FFT spectrum in inset indicates a good uniformity of the pure microlens array. **b**, Virtual image array of crosses (+) obtained by the pure microlens array. The corresponding FFT spectrum in the inset and the clear virtual image of crosses (+) reflect that each lens has a similar size and shape, further indicating the good uniformity of the pure lenses. **c**, Cross-section of the pure microlens array along inscribed circle diameter (ID), showing 4.5 µm-diameter and 150 nm-height of these concave microlenses. **d**, Cross-sections of a microlens along the circumscribed circle diameter (CD, blue) and the inscribed circle diameter (ID, red) well overlap, showing that the microlenses are almost isotropic and spherical.

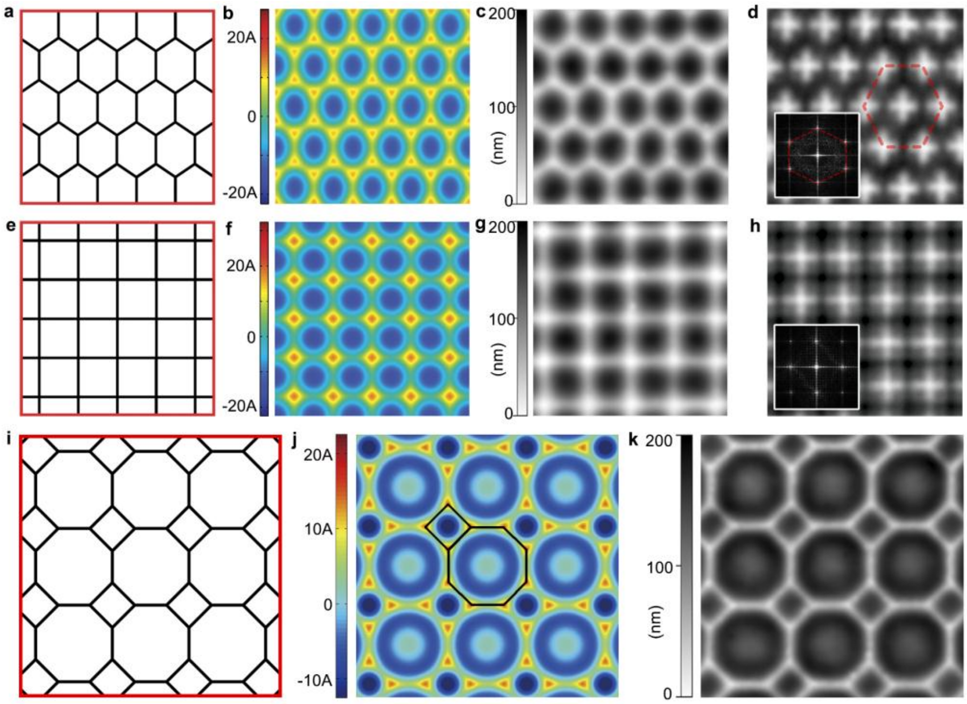

Supplementary Figure 7 | Microlens arrays with special shapes. a,e,i, LiPs with the configurations of oval-hexagons (4 μm×5.6 μm, elongation ~ 20 %), squares (4.5 μm × 4.5 μm) and dual-shaped pattern consisting of octagons (9 μm × 9 μm) and squares (3.7 × 3.7 μm). **b,f,j,** Calculated results of oval-hexagonal, square and dual-shaped microlens arrays by superposition of LiDs. **c,g,k,** Experimental height images of oval-hexagonal, square and dual-shaped microlens arrays. Here the experimental topographies are obtained by the laser scanning confocal microscope. **d**,**h**, Corresponding imaging performances of oval-hexagonal and square microlens arrays. It is easily to see that from FFT spectra in the insets of **d** and **h,** each lens in the hexagonal array (**c**) is elongated and each lens in the square array (**g**) is regularly tetragonal, matching with their configurations. Image size: **a-h**, 20 μm × 20 μm; **i-k**, 30 μm × 30 μm.

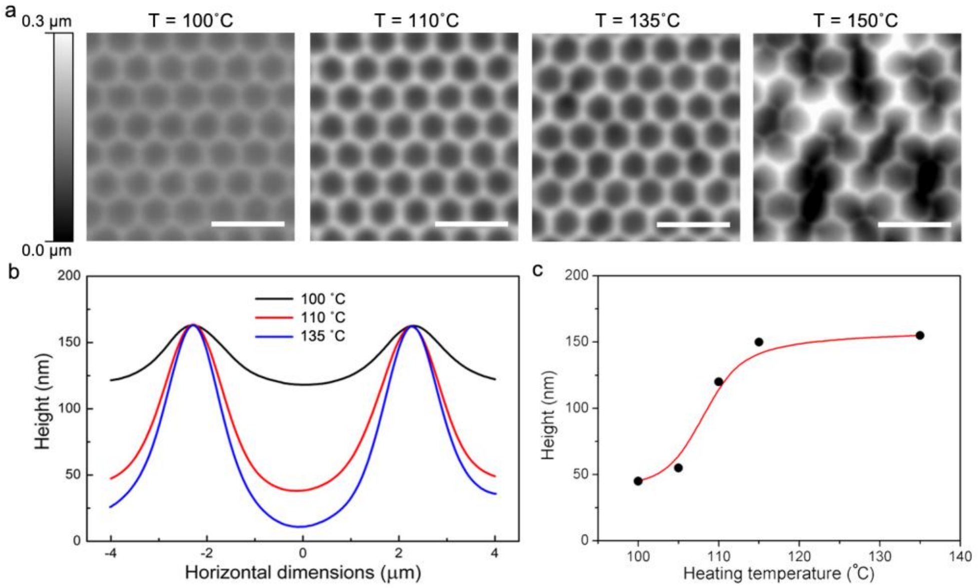

Supplementary Figure 8 | Tunable height of microlens array. **a**, Height images of pure microlens arrays at different heating temperatures (*T*). Scale bars: 10 μm. To reach the critical thermal strain, 100 ˚C is the lowest temperature of generating microlens arrays induced by LiPs. At such a lower temperature, the formed lens structure is akin to ripples with a height of ~25 nm, which is too low for applications. With the increase in heating temperature from 110 ˚C to 135 ˚C, the height rises from 50 nm to 150 nm. However, when an excessively high temperature is applied, for instance, 150 ˚C or higher, the microlens array will be damaged, which might be attributable to the viscosity of PS at such high temperature. Here the experimental topographies are obtained by using a laser scanning confocal microscope. **b,** Corresponding cross-sectional profiles of the pure microlenses with the same size and tunable height at different heating temperatures. **c,** Relationship between the height of the microlenses (with the same size) and heating temperature.

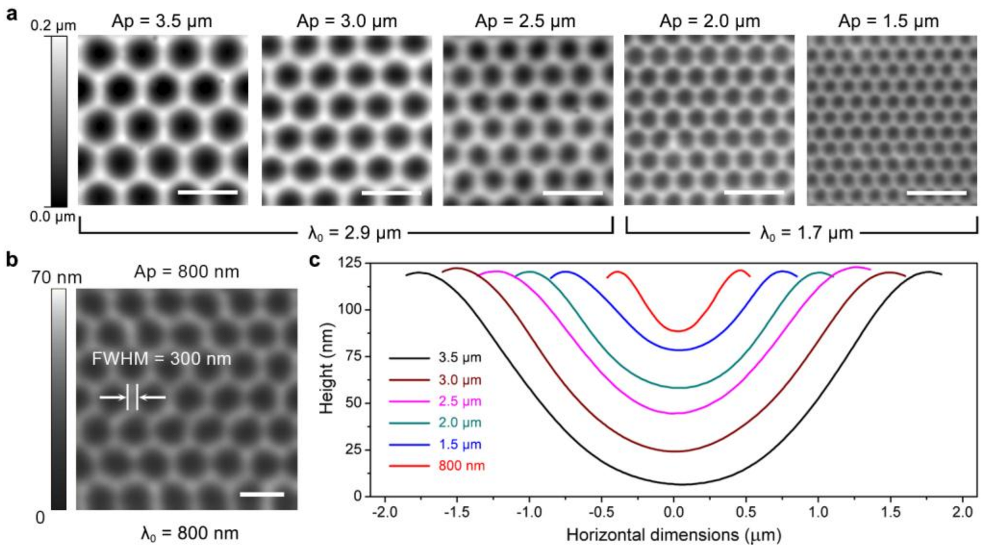

Supplementary Figure 9 | Tunable microlens array in size. a, Microlens arrays with unchanged shape and curvature for different apertures (Ap). The two microlens arrays on the right (Ap = 1.5, 2.0 μm) are made in an Au/PS bilayer with λ_0 = 1.7 μm and the rest (Ap = 2.5, 3.0, 3.5 μm) is in another Au/PS bilayer with λ_0 = 2.9 μm, indicating the high-quality microlens arrays with the same profile but different apertures. Here the experimental topographies were obtained by using laser scanning confocal microscope. Scale bars: 5 μm. **b,** A minimum lens with Ap of 800 nm can be made in a Au (3 nm)/PS (100 nm) bilayer (λ_0 = 800 μm). This experimental topography was obtained by AFM. The full width at half maximum (FWHM) in this case is about 300 nm, close to the focused laser spot size. Scale bars: 500 nm. **c,** Corresponding cross-sections of microlenses in **a** and **b** with the same profile but different sizes.

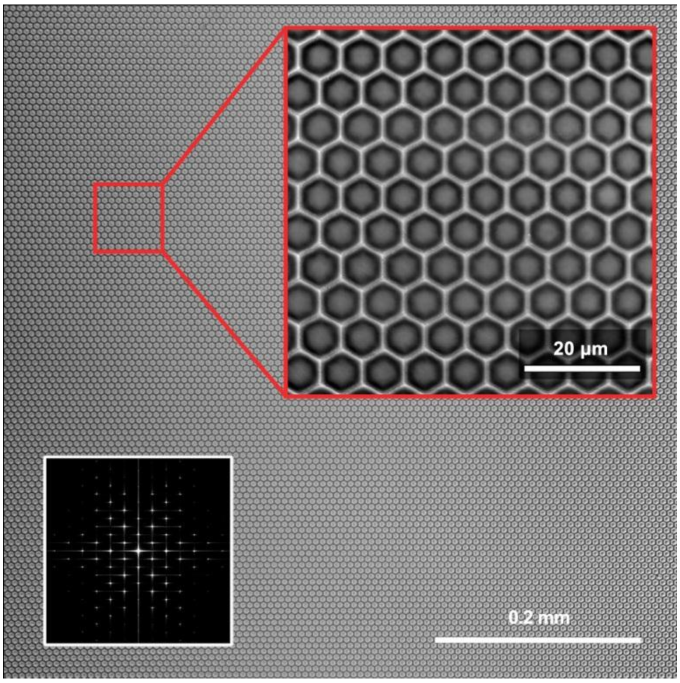

Supplementary Figure 10 | Large-area convex-concave microlens array. Laser scanning confocal microscopy images shows a large-area convex-ooncave microlens array. Magnified topography is shown in the inset at the top-right corner. Here the ID of convex-concave microlens array is 7 μm and the λ_0 of the PS/Au bilayer is 2.9 μm. The FFT at the bottom-left corner shows that in the large-area convex-concave microlens array, all lens cells have a good uniformity without defects. It should be noted that in principle, wafer-scale or even larger area can be realized as long as the moving stage in LDW equipment is large enough.

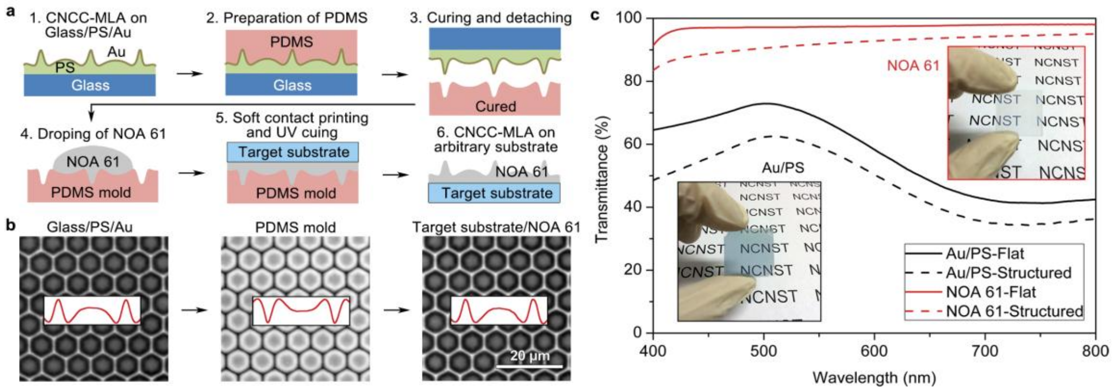

Supplementary Figure 11 | Replication and transfer of convex-concave microlens array to targeted substrates. a, Schematic illustration of the procedure for replicating with different materials and transferring convex-concave microlens array to targeted substrates (see details in Method). **b**, The **i**mages of laser scanning confocal microscope and corresponding cross sectional profile of the lens structures in Au/PS bilayer, PDMS mold, and NOA 61, indicating an accurate replication and transfer. **c**, The transmittance spectrum of Au/PS and NOA 61 in visible range shows that the convex-concave microlens array has been transferred from a semitransparent bilayer to a transparent material. The high transparency of convex-concave microlens array ensures its possible applications in solar cells and light emitting diodes without worrying about a serious loss of light energy.

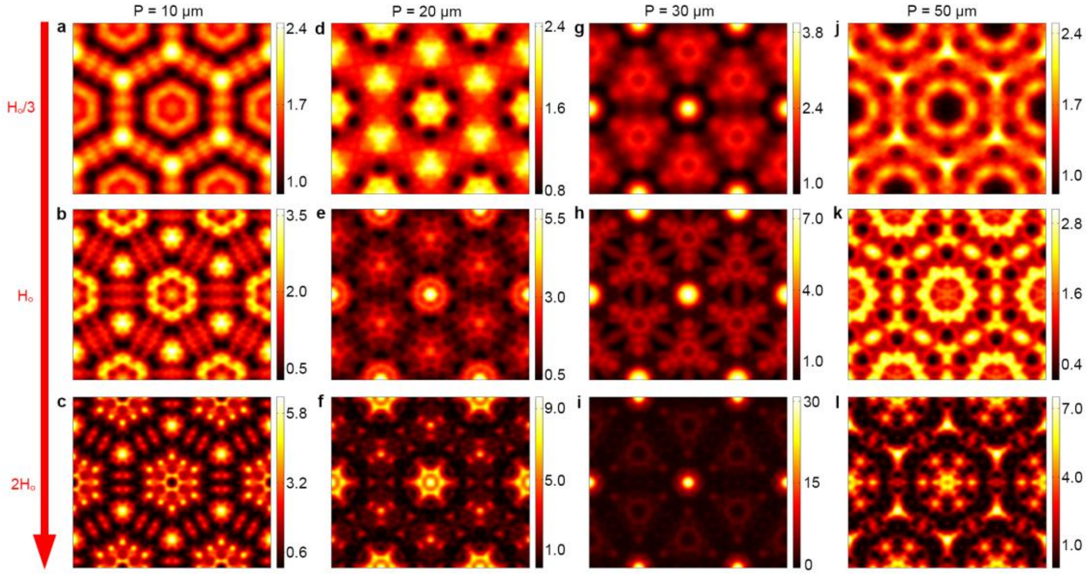

Supplementary Figure 12 | Simulated intensity distribution remarkably changes with different relief heights and different *z*-positions. Image area: 20 μm × 17.3 μm; color bar: normalized intensity; H_o: original experimental height of **c**onvex-concave microlens array. To reveal the influence of the relief height to the imaging patterns, in our FDTD simulation, we select three different values of relief height, which are $H_o/3$ (**a, d, g, j**), H_o (**b, e, h, k**) and $2H_o$ (**c, f, i, l**), respectively. For the three cases, we all show four simulated images for four different positions (P = 10, 20, 30, 50 μm). Obviously, imaging pattern at the same position is quite different for different relief height of the convex-concave microlens array. At P = 30 μm, the patterns are not that different because of the focusing effect of the convex-concave microlens array. This result indicates that the relief height of the convex-concave microlens array has a significant effect on imaging pattern.
